# Burden of Sickle Cell Disease in Ghana: The Korle-Bu Experience

**DOI:** 10.1155/2018/6161270

**Published:** 2018-12-02

**Authors:** Eugenia V. Asare, Ivor Wilson, Amma A. Benneh-Akwasi Kuma, Yvonne Dei-Adomakoh, Fredericka Sey, Edeghonghon Olayemi

**Affiliations:** ^1^Ghana Institute of Clinical Genetics, Korle-Bu, Accra, Ghana; ^2^Department of Haematology, Korle-Bu Teaching Hospital, Accra, Ghana; ^3^Department of Haematology, College of Health Sciences, University of Ghana, Accra, Ghana

## Abstract

In Africa, sickle cell disease (SCD) is a major public health problem with over 200,000 babies born per year. In Ghana, approximately 15,000 (2%) of Ghanaian newborns are diagnosed with SCD annually. A retrospective review of medical records of all SCD patients aged 13 years and above, who presented to the sickle cell clinic at Ghana Institute of Clinical Genetics (GICG), Korle-Bu, from 1st January 2013 to 31st December 2014, was carried out, using a data abstraction instrument to document their phenotypes, demographics, attendance/clinic visits, pattern of attendance, and common complications seen. During the period under review 5,451 patients were seen at the GICG, with 20,788 clinic visits. The phenotypes were HbSS (55.7%) and HbSC (39.6%) with other sickle cell phenotypes (4.7%). Out of the 20,788 clinic visits, outpatient visits were 15,802 (76%), and urgent care visits were 4,986 (24%), out of which 128 (2.6%) patients were admitted to the Teaching Hospital for further management of their acute complications. There were 904 patient referrals (out of 5,451 patients) for specialist care; the 3 specialties that had the most referrals were Obstetrics and Gynaecology (168 patients), Orthopaedics (150 patients), and Ophthalmology (143 patients). In 2014, complications seen at KBTH included 53 patients with avascular necrosis (AVN) and 61 patients with chronic leg ulcers. Our centre has a large number of patients living with sickle cell disease. From our experience, early recognition and referral of sickle cell related complications can reduce morbidity and mortality associated with this disease. A multidisciplinary approach to care of SCD patients is therefore important.

## 1. Introduction

Genetic diseases are very common; and it has been estimated that more than 7 million babies are born each year with a congenital genetic abnormality [1].

Sickle cell disease (SCD) is the most common haemoglobinopathy [1]; it is characterized by inheritance of 2 abnormal haemoglobins of which one is haemoglobin S (HbS). Haemoglobin S (HbS) is a structural variant of normal adult haemoglobin (HbA), inherited as an autosomal recessive Mendelian trait. The most common clinical phenotype is the homozygous form (HbSS or sickle cell anaemia). Compound heterozygous SCD include HbSC, HbSD, HbSO-Arab, and HbS/beta-thalassemia. Heterozygotes are generally less symptomatic compared to those who are homozygous [2].

Sickle cell disease is a major public health problem with over 200,000 babies born per year with SCD in Africa [3, 4]. Approximately 80% of all children born with SCD are in sub-Saharan Africa [1, 5]. In Ghana, 2% (about 15,000) of newborns have SCD, with 55% of them having the homozygous form [6].

Clinical features of SCD include acute pain episodes (which are the hallmark of the disease), anaemia, recurrent infections, and chronic end-organ damage [7, 8]. Newborn screening with early diagnosis and comprehensive care [9–16] has been shown to improve survival since the disease has a high mortality rate in the first few years of life. In 2010, Quinn et al. reported an increased life expectancy in the American SCD population, with over 90% of babies born with SCD currently reaching adulthood [17].

Despite the high prevalence of SCD in Ghana, the extent of the burden of the disease in adults is yet to be quantified and the life expectancy of the Ghanaian SCD patient is not known, though it is generally agreed that more children with the disease now survive into adulthood. Many of the newer modalities of management such as hydroxyurea are not widely used.

The Ghana Institute of Clinical Genetics (GICG) was established in Korle-Bu, Accra, Ghana, in 1974 and currently provides comprehensive outpatient care to both adolescents and adults with SCD, along with community education and research. The premier adult sickle cell clinic in Ghana is located in the institute with the largest number of registered adolescent and adult SCD patients in Ghana. The clinic receives patients (adolescents and adults) from all over Ghana but mainly from the southern part of the country.

This study was designed to outline the burden of sickle cell disease at the GICG and identify the common complications.

## 2. Materials and Methods

### 2.1. Study Design

A retrospective two-year chart review of all patient folders and records from January 1st, 2013, to December 31st, 2014, was carried out [2]. Institutional approval was obtained from GICG.

### 2.2. Study Sites

The GICG located on the campus of Korle-Bu Teaching Hospital renders outpatient services through an outpatient department and an urgent care unit. Referrals are received from other healthcare facilities all over Ghana. It has over 25,000 registered SCD patients. Every year, the clinic records between 10,000 and 15,000 clinic visits with an average daily attendance of almost 50 patients. Patients who need further specialist care are referred to the Teaching Hospital.

### 2.3. Study Population

The study population was made up of all SCD patients aged 13 years and above who presented to GICG and KBTH within the study period.

### 2.4. Data Collection

The demographic characteristics, clinical information, and pattern of attendance were obtained from the case files of all eligible patients. A data extraction form was used to document demographic characteristics and clinical information such as age, sex, sickle cell phenotypes, and sickle cell related complications (the data on SCD complications was extracted from the following departments at the Korle-Bu Teaching Hospital: Obstetrics and Gynaecology, Orthopaedics, Ophthalmology, and Urology). The World Health Organization (WHO) age group classification was used as follows: adolescents from 10 to 19 years, adults from 20 to 59 years (young adults from 20 to 39 years; middle age from 40 to 59 years), and elderly from 60 years and above [18]. The age group from 15 to 44 years is considered the reproductive age [18]. For the purposes of this study, our adolescent age group started from 13 to 19 years, because at our centre patients below 13 years are seen in the paediatric department.

### 2.5. Data Storage and Management

The data collected from the medical records was limited to only information that was necessary to the study. No personal identifiable data was collected. Only the authors had access to the data.

### 2.6. Data Analysis

Data were captured using Microsoft Access 2010 version, analysed using Excel (windows version 10), reported with simple descriptive statistics such as proportions, ratios, percentages, tables, and histograms.

## 3. Results

### 3.1. Phenotypic Patient Burden at GICG

**Figure 1:**
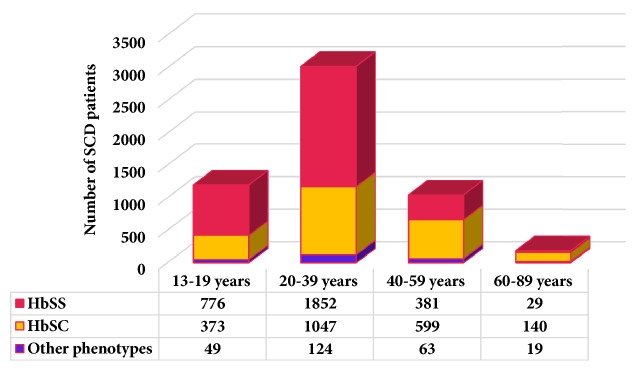
Age group and phenotypes of SCD patients.

Over the period of review, 5,451 adolescent and adult SCD patients were seen at the study site, with 20,788 clinic visits. The phenotypes were HbSS (55.7%), HbSC (39.6%), and other sickle cell phenotypes (4.7%). The male-to-female ratio was 1:1.6. The ages of patients seen at the clinic during the review ranged from 13 to 87 years with a higher proportion of young adults and middle-aged patients (Figure 1). A third (1,400) of the patients were in the reproductive age group. From age 13 to 44 years, there were more HbSS patients as compared to HbSC (ratio of 2:1). However, this was reversed after the age of 44 years.

### 3.2. Clinic Visits

**Figure 2:**
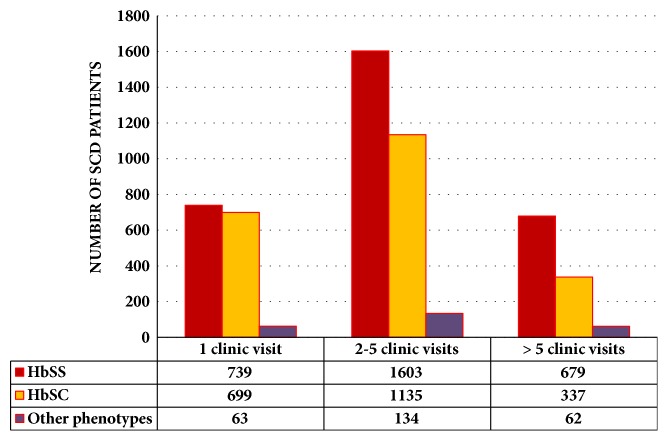
Clinic visits by phenotypes.

Over the two-year study period, there were 20,788 clinic (GICG) visits made by the SCD patients. Approximately 27.5% of the patients made one clinic visit, 52.7% made 2 to 5 clinic visits, and 19.8% made >12 clinic visits per year (Figure 2).

Patients with HbSS phenotype were responsible for 61% of the clinic visits compared with 34% for HbSC and 5% for other phenotypes.

Clinic attendance was highest in January (approximately 1000) and lowest in December (approximately 700), with another increase seen from early May to late July (Table 1).

### 3.3. Proportion of SCD Patients Who Had Further Specialist Care at KBTH

During the study period, out of 5,451 patients seen, 904 (16.6%) were referred for specialist care at the Teaching Hospital (Table 2). The three specialties that had the most referrals were the Obstetrics and Gynaecology clinic (168 patients), the Orthopaedic clinic (150 patients), and the Ophthalmology clinic (143 patients).

### 3.4. Common Complications Confirmed in Patients Referred for Specialist Care

Records from the Orthopaedics department, KBTH, in 2014 showed that 53 (68.8%) out of 77 SCD patients seen were diagnosed with radiological evidence of avascular necrosis (AVN). Most patients were diagnosed between the ages of 20-24 years. Forty-nine (92.5%) of these had AVN of the femoral head and 4 (7.5%) had AVN of the humeral head. Only 4 of them had bilateral AVN of the femoral head.

At the Ophthalmology department, 16 (18.6%) out of the 86 patients seen in 2014 were diagnosed with sickle cell retinopathy. Twenty-eight (51.9%) of 54 patients seen by the urologists in 2014 had priapism.

### 3.5. Proportion of SCD Patients Seen at GICG with Chronic Leg Ulcers

At the end of 2014, 61 SCD patients were seen at the GICG with chronic leg ulcers who were referred to either the general surgical or plastic surgery units. Chronic leg ulcers were more common in the male sex and phenotype SS and were mostly unilateral.

## 4. Discussion

Sickle cell disease is a major public health problem in Africa, where over 200,000 babies are born with the disease per year [3, 4]; and about 80% of all children born with SCD are in sub-Saharan Africa [1, 5]. There were more patients with HbSS compared to HbSC in our study (55.7% versus 39.6%) with a higher female-to-male ratio (1.6:1). This agrees with an earlier study by Ohene-Frempong et al. (2008) from Ghana, which showed that 55% of children born with SCD in Ghana had HbSS [6]. From ages of 13 to 44 years, the ratio of HbSS to HbSC was 2:1; this was reversed after 44 years possibly as a result of the higher mortality seen in HbSS patients who have been documented to have a more severe form of the disease [19].

The slightly higher female-to-male ratio in our study may be due to the better health seeking habits of females as compared to males [20] and the fact that in most populations women live longer than men [21]. It is therefore not surprising to see that the women in our cohort had better health maintenance, judging by their attendance. In Ghana, according to WHO data published in 2015, the life expectancy (in years) at birth for the Ghanaian male is 61.0 and the Ghanaian female is 63.9 [21]. This may also contribute to the male-to-female ratio of 1:1.6 seen in this cohort.

Expectedly, HbSS patients accounted for more clinic visits (61%) than other SCD phenotypes, since they are known to have a more severe form of SCD [19].

The effect of the Ghanaian climate was also seen in the pattern of clinic attendance by our patients. Ghana has a tropical climate; temperature in the country varies with season and elevation. In the Southern part of the country where Accra is located, two rainy seasons occur, from April to July and from September to November. The Harmattan, a dry desert wind, blows from the northeast from December to March. The Harmattan lowers humidity, creating hot days and cool nights in the north. In the south, the effects of the Harmattan are felt in January. In most parts of Ghana, the highest temperatures occur in March and the lowest in August [22].

Clinic attendance was lowest in December, probably because of the festive season, and highest after the festive season in January, which is often the peak of Harmattan season with cold, dry conditions, which predisposes SCD patients to developing crises. There was another peak from May to July as a result of the frequent rainfall, cold, and very humid weather conditions; these along with the increase in incidence of malaria [23] may also predispose our patients to ill-health and crises.

Unpublished data from KBTH shows that approximately 200 pregnant women with SCD are seen at the antenatal clinic each year. Despite the well-documented high rates of maternal and foetal morbidity and mortality in pregnant women with SCD [24], there is still a paucity of preconception care or family planning in this population [25, 26]. Given these pregnancy-associated problems for women with SCD, advice about both pregnancy planning and effective contraception is of paramount importance [27].

Our data showed that over 900 patients were referred for further specialist's care and that three specialties (Obstetrics and Gynaecology, Orthopaedics, and Ophthalmology) had over 50% of the referrals. With improved care, more children with SCD now survive into adulthood and are now prone to chronic complications which are more common in adults such as avascular necrosis and retinopathies.

## 5. Conclusions

Our study confirms that Ghana has a large burden of SCD; a pilot newborn screening program in one of the ten regions of Ghana has shown prevalence of 1.8%, which translates to about 15,000 babies with SCD being born in Ghana every year [6]. It is likely that, with appropriate use of basic medical facilities, more children with SCD now survive into adulthood with the oldest patient in our cohort now in her late 80s. It is almost certain that if Ghana and other African countries are to make an appreciable impact on the care of people living with SCD, more attention has to be paid to providing multidisciplinary care including adequate care at the primary level along with the development and implementation of a national sickle cell disease policy which will include but will not be limited to universal new born screening [28].

## Figures and Tables

**Table 1:** Pattern of attendance at GICG (2013-2014).

	**2013**						**2014**					
**Month**	**HbSS**	**HbSC**	**Other**	**Total**	**New patients**	**D.A.A**	**HbSS**	**HbSC**	**Other**	**Total**	**New patients**	**DAA**
**January**	620	350	56	1026	25	46.64	656	333	55	1044	16	47.45
**February**	540	294	48	888	24	44.40	492	299	33	825	12	41.25
**March**	514	240	38	790	36	41.58	517	296	29	854	19	42.70
**April**	330	173	27	530	7	29.44	468	275	39	783	16	39.15
**May**	551	275	42	868	20	41.33	532	324	41	898	14	44.90
**June**	537	311	69	919	16	45.95	488	312	46	849	16	40.43
**July **	621	364	29	1017	27	46.23	523	276	41	842	13	40.10
**August**	600	343	49	993	28	47.29	578	316	46	940	16	44.77
**September**	576	307	25	908	13	45.40	531	343	43	919	15	43.76
**October**	577	342	39	958	21	43.55	443	262	32	737	15	33.50
**November**	550	313	51	913	24	43.48	492	272	36	804	14	40.20
**December**	478	269	40	787	10	41.42	427	241	29	696	16	34.80
**Total**	**6494**	**3581**	**513**	**10597**	**251**	**43.06**	**6147**	**3549**	**470**	**10191**	**182**	**41.08**

DAA: Daily Average Attendance.

**Table 2:** SCD patients referred for specialist care.

**Specialty**	**2013**	**2014**	**Total**
**Obstetrics**	77	91	168
**Orthopaedics**	75	75	150
**Ophthalmology**	70	73	143
**Plastics/general surgery**	20	53	73
**Urology**	27	21	48
**Nephrology**	9	12	21
**Others**	153	148	301
**Total**	**431**	**473**	**904**

## Data Availability

The data used to support the findings of this study are available to researchers who meet the criteria for access to confidential data from the corresponding author upon request. This will be done after approval of the request by the management committee, Ghana Institute of Clinical Genetics.
